# Genomic Insights into Lipid Dependency in Atypical Strains of *Malassezia pachydermatis*

**DOI:** 10.1007/s11046-025-01039-0

**Published:** 2025-12-22

**Authors:** Leyna Díaz, Gemma Castellá, Riccardo Aiese Cigliano, Walter Sanseverino, F. Javier Cabañes

**Affiliations:** 1 Grup de Micologia Veterinària, Departament de Sanitat i d’Anatomia Animals, Facultat de Veterinària, Universitat Autònoma de Barcelona, 08193 Bellaterra, Catalonia Spain; 2 Sequentia Biotech SL, Barcelona, Catalonia Spain

**Keywords:** *M. pachydermatis*, Genome, Resequencing, Lipid dependency

## Abstract

The yeasts of the genus *Malassezia* are part of the normal skin microbiota of a wide range of warm-blooded animals including humans. Within this genus, *Malassezia pachydermatis* is commonly found in the normal skin microbiota of a variety of animal hosts. *Malassezia* yeasts are considered lipid-dependent due to their inability to synthesize long chain fatty acids de novo. While *M. pachydermatis* is typically able to grow on Sabouraud’s agar (SGA) without lipid supplementation, certain strains display an atypical lipid dependency and are unable to grow on SGA. The aim of this work was to study the genomic differences between atypical *M. pachydermatis* strains unable to grow on SGA and the reference strain. The genomes of three atypical lipid-dependent *M. pachydermatis* strains were sequenced using Illumina technology and compared with the reference genome of *M. pachydermatis* neotype strain CBS 1879. A total of 397 small variants with a high or moderate impact on the protein were observed in genes involved in lipid metabolism. Of those small variants observed we highlight the ones observed in 12 out of the 13 genes encoding secretory lipases and in the *CKI1* gene that is unique to *M. pachydermatis* within the genus. The analysis of those small variations suggested a variation in their ability to adapt to environmental changes and their requirements to grow in different culture media.

## Introduction

The yeasts of the genus *Malassezia* are part of the normal skin microbiota of a wide range of warm-blooded animals including humans [1, 2]. The genus includes 20 species, some of which have been isolated from various animal hosts while others tend to be more host specific as *M. vespertilionis* isolated from bats [3], *M. cuniculi* isolated from rabbits [4] or *M. gallinae* isolated from chickens [5]. Even though they are considered commensals, under certain circumstances some of these species may transition to opportunistic pathogens causing common skin disorders [2].

Lipid metabolism plays a crucial role in *Malassezia*’s metabolism. *Malassezia* yeasts can metabolize fatty acids to carry out a variety of biological processes such as energy homeostasis and storage, molecular signalling, membrane biogenesis, mediation of cell apoptosis and fusion, metabolism and even contribute to their pathogenicity [6, 7]. However, *Malassezia* species are unable to synthetize long-chain (C14-C16) fatty acids de novo due to the lack of the gene encoding for the fatty acid synthase [8, 9]. Therefore, they rely on the environment as a source of fatty acids. For this reason, *Malassezia* yeasts secrete several hydrolases such as lipases, esterases and phospholipases to supply their lipid requirements. It has been observed an expansion of phospholipase and secretory lipase gene families in *Malassezia*’s genome, especially in *M. pachydermatis*, where a high number of lipase gene gain events have occurred [8, 10]. The released fatty acids are then metabolized and modified by other lipid metabolism enzymes. They can then be used for the synthesis of triglycerides and sterols, for the synthesis of essential membrane lipids or can be degraded by β-oxidation enzymes [11]. This loss and gain of genes are possibly a consequence of the adaptation of these yeasts to the host’s skin and mucosae which provide at least lipids containing the essential fatty acids required for their growth [8, 12]. Therefore, for the isolation of *Malassezia* species enriched culture media with a specific lipid composition such as modified Dixon agar (mDA) [13] or Leeming and Notman agar (LNA) [14] are necessary. There is some variability in the lipid-dependency among species of the genus, thus conferring them different metabolic versatility [10, 15]. In fact, *M. pachydermatis*, was considered the only *Malassezia* species non-lipid-dependent due to its unique ability to grow on Sabouraud’s agar (SGA) [16]. Nevertheless, genomic analysis revealed that *M. pachydermatis’* genome also lacks fatty acid synthase but, it is uniquely able to utilize lipid fractions within the peptone component of SGA [8, 9]. On the other hand, several *M*. *pachydermatis* isolates from dogs have shown some inconsistent lipid dependence and were able to grow on SGA after some subsequent transfers on this medium [17]. However, a few *M*. *pachydermatis* strains unable to grow on SGA have been reported [18–20]. In our laboratory, we characterized three lipid-dependent *M*. *pachydermatis* strains isolated from domestic animals [20]. The finding of these peculiar strains exemplifies the diversity within *M*. *pachydermatis* which involves atypical strains with particular growth requirements.

The aim of this work was to study the genomic differences between atypical *M. pachydermatis* strains unable to grow on SGA and the reference strain. To assess these growth limitations, the genomes of three atypical lipid-dependent *M. pachydermatis* strains were sequenced using Illumina technology and compared with the reference genome of *M. pachydermatis* neotype strain CBS 1879. We focused on the analyses of structural and small variants common to these atypical strains that were involved in lipid metabolism.

## Materials and Methods

### Strains

Three *M. pachydermatis* haploid strains (MA366, MA374 and MA380) from our collection were studied. These strains were described as atypical lipid-dependent *M. pachydermatis* strains as they were unable to grow on SGA without lipid supplementation. Their lipid-dependency has been previously evaluated, and their identity has been confirmed by DNA sequencing [20]. The three strains were isolated from the external ear canal of healthy animals. Strain MA374 was isolated from a cow and MA366 and MA380 were isolated from dogs.

### *Malassezia* Culture and DNA Extraction

The three atypical *M. pachydermatis* strains were cultured on modified SGA supplemented with 4 ml/L Tween 40 and 1 ml/L Tween 20, for 4–5 days at 35 °C. Genomic DNA extraction for Illumina sequencing was done with a phenol:chloroform-based protocol previously described [21]. The eluted DNA was stored at − 20 °C until used.

### Genomic DNA Sequencing and Variant Analysis

The three *M. pachydermatis* strains (MA366, MA374 and MA380) were sequenced using an Illumina NovaSeq 6000 machine to produce short-paired end reads of 150 bp after the generation of libraries using the Illumina TruSeq PCR Free protocol. Prior to further analysis, a quality check was performed on the raw sequencing data, removing low quality portions while preserving the longest high-quality part of NGS reads. A minimum length of 35 bp and a quality score of 25 were set using BBDuk software [22]. The resulting high-quality reads were mapped against the *M. pachydermatis* reference genome downloaded from the NCBI database (GCF_001278385.1) together with the genome annotation. The mapping was performed with the software minimap2 [23] with the -x sr option. The resulting BAM files were sorted and filtered to remove optical duplicates using the software sambamba [24].

The identification of small variant sites (variant calling; VCs) was performed with the software Freebayes [25] using only uniquely mapping reads (MAPQ > 30), genotyping sites with a coverage of at least 10X and setting a ploidy of 1. In the following step, VCFtools [26] was used to keep only variants with a quality higher than 30 and a read depth of at least 30X. The identification of structural variant sites (SVs) as big deletions, insertions, inversions, duplications and translocations was performed using the MANTA algorithm [27]. The SVs are identified using an approach based on the distance of the paired-end and on the detection of split reads. The obtained variants were then filtered to include only those with a minimum quality of 30 and a minimum depth of 30X. The filtered variants were annotated using the software VEP (Variant Effect Predictor) [28]. During the annotation step, the effect of each variant on the genes in the genome was predicted by the software VEP to infer the presence of low, moderate, or high impact variants.

## Results

### Resequencing Study

The general resequencing genome data of the three atypical *M. pachydermatis* strains is summarized in Table 1. On average 95% of the reads passed the initial quality control. Thus, we obtained a mean of 16,904,854 reads per strain, of which 95.25% (16,095,120) mapped on the reference genome with a depth coverage between 264 and 322X. The GC content of the genomes ranged from 52.17 to 52.65%. The resulting mapping of the reads from the atypical *M. pachydermatis* strains to the CBS1879 reference genome allowed the study of the genomic differences that could explain the lack of growth in SGA. The genome resequencing information of *M. pachydermatis* MA366, MA374, and MA380 have been deposited in the NCBI Sequence Read Archive (SRA) database under accession PRJNA1293882.

**Table 1:** Statistics of resequencing results

	MA366	MA374	MA380
Raw data	8,556,457	10,002,459	8,080,955
Trimmed data	7,985,743	9,617,819	7,753,719
% Passed	93.33	96.15	95.95
Number of reads	15,971,486	19,235,638	15,507,438
Mapped reads	15,220,961	18,206,465	14,857,935
% Mapping	95.3	94.65	95.81
Average coverage (X)	269	322	264
% GC	52.17	52.65	52.47

### SV Analysis

**Fig. 1:**
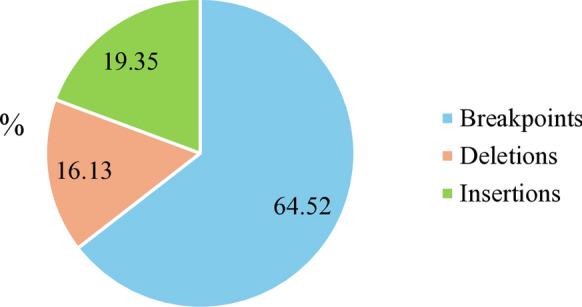
Structural variant types observed common to the three strains

**Fig. 2:**
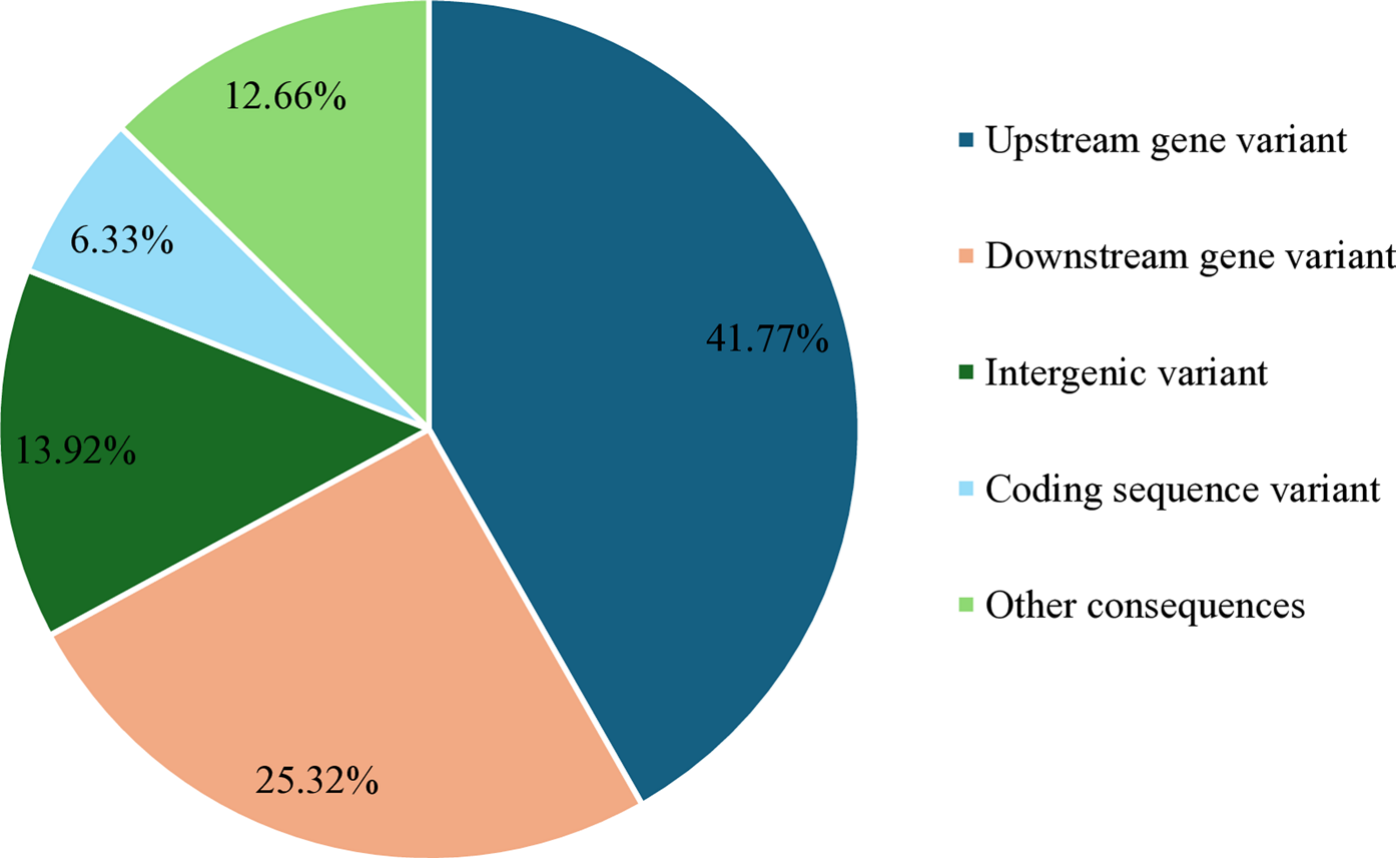
Most severe consequences common to the three strains produced by structural variants

A total of 279 different SVs were observed across the three strains from which 31 were common to the three strains (Table 2). Of those common SVs, 64.52% were breakpoints, 19.35% insertions and 16.13% were deletions (Fig. 1). Focusing on their effect, 41.77% were upstream gene variants, 25.32% were downstream gene variants, 13.92% were intergenic variants, and 6.33% were coding sequence variants (Fig. 2). For further analysis we only focused on those SVs that were common to the three strains and produced a moderate or high impact. Therefore, six SVs with a moderate or high impact were found common to the three strains and were distributed into six different genes (Table 3). Although none of those were related to lipid metabolism, a high impact 1,092 bp deletion was identified. The rest of the common SVs observed had a low impact or an undefined impact on the resulting protein.

**Table 2:** Structural variant analysis summarized per strain

Structural variants (SV)	MA366	MA374	MA380
Total SVs	198	147	312
Number of insertions	67	47	108
Number of deletions	90	72	157
Number of breakpoints	40	26	44
Number of duplications	1	2	3
Different SVs		279	
Common SVs		31

**Table 3:** Common structural variants observed

ID	Gene description	Function	Impact type	VEP prediction
2872812	Protein kinase	Intracellular signal transduction, microtubule cytoskeleton organization and protein phosphorylation	Moderate DEL (− 55 bp)	Protein altering variant
28730611	Nucleoside	Transmembrane transporter activity	Moderate INS (228 bp)	Protein altering variant
28730102	Tripeptidyl peptidase	Serine-type endopeptidase activity and tripeptidyl-peptidase activity	High DEL (− 1092 bp)	Stop lost, coding sequence variant, 3 prime UTR variant and feature truncation
28728297	DEAD-domain-containing protein	rRNA processing	Moderate DEL (− 24 bp)	In-frame deletion
28726826	Histone-lysine n-methyltransferase SETD1	Nucleic acid binding, histone H3K4 methyltransferase activity and protein binding	Moderate INS (29 bp)	In-frame insertion
28730018	Essential component of the nuclear pore	Nuclear import and export	Moderate DEL (− 66pb)	In-frame deletion

### VC Analysis

**Fig. 3:**
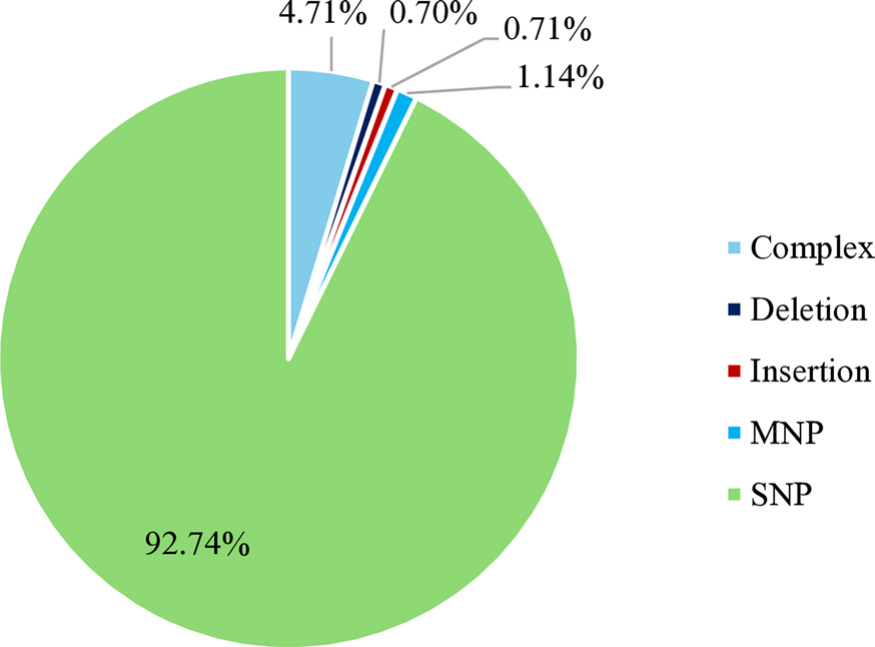
Small variant types observed common to the three strains

**Fig. 4:**
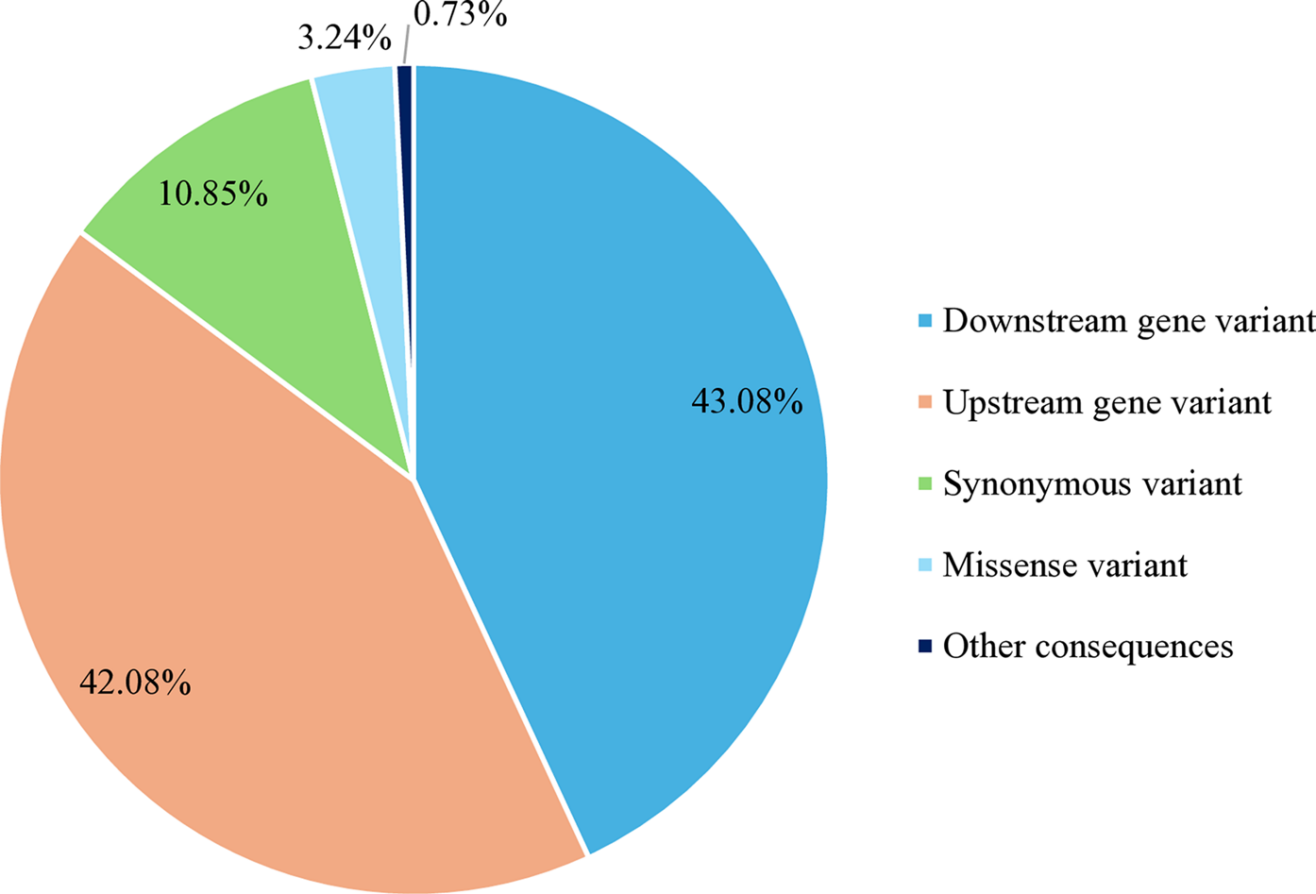
Most severe consequences produced by small variant sites common to the three strains

A total of 344,667 different small variants were observed across the three strains, of which 65,672 variants were detected common to the three strains (Table 4). Of those common small variants, 92.74% were single nucleotide variants, 4.71% were complex substitutions, 1.4% were indels and 1.14% were multiple nucleotide polymorphisms (Fig. 3). Focusing on their effect, 43.03% were downstream gene variants, 42.08% upstream gene variants, 10.85% were synonymous variants and 3.24% were missense variants (Fig. 4). Regarding their impact on the protein, 5,097 small variants with a moderate or high impact were found common to the three strains and were distributed into 1,383 genes. For further analysis, this study only focused on common small variants that had a moderate or high impact on the protein and were related to lipid metabolism. Therefore, a total of 397 small variants were observed in genes involved in lipid metabolism (Table 5). Only two small variants showed a high impact on the resulting protein, producing the loss of a start and a stop codon, respectively. Both high impact SNPs were found in genes encoding secretory lipases TGL3 (ID 28726643 and ID 28728420). The remaining alterations produced a moderate impact on the protein and were considered missense variants. Only two of these moderate impact small variants were splice region missense variants. These 397 small variants were distributed into 99 genes, of which 23% were lipases and esterases, 11% were genes related to sterol metabolism, 33% were genes involved in phospholipid metabolism and 20% were genes of fatty acid metabolism. Also, variants were observed in other genes related to the lipid metabolism (12%) as transferases or lipid binding enzymes. Within the group of lipases (Table 5), 23 lipase-like genes including secretory lipases and esterase lipases were found to have 73 moderate and 2 high impact small variants altering the protein function. A total of 12 genes encoding secretory lipases showed moderate and high impact small variants. Also, several esterase and esterase lipase encoding genes were altered. Within the group of sterol synthesis genes, 11 essential genes such as the *ERG2, ERG5, ERG9, ERG10, ERG13, ERG24* or the *HMG1/2* genes, related to sterol and ergosterol synthesis showed 32 moderate impact small variants. Regarding the group of genes involved in phospholipid metabolism, 33 genes of importance for the synthesis of phospholipids such as phospholipases, the *CHO2* gene and the *CKI1* gene showed 163 alterations. Nevertheless, 20 essential genes involved in fatty acid metabolism showed 86 moderate impact small variants. Within this group of fatty acid metabolism genes, the alterations observed in the Δ-9 and Δ-12 desaturase genes, the Acyl-CoA oxidase gene, the Acetyl-CoA synthetase gene and both copies of the polyketide gene stand out due to their relevance for the lipid metabolism. A total of 44 small variants were also observed in other 12 genes involved in lipid metabolism, such as genes related to lipid binding, homeostasis and other metabolic processes.

**Table 4:** Small variant analysis summarized per strain

Small variants	MA366	MA374	MA380
Total small variants	220,731	221,029	224,711
Number of SNPs	169,552	169,690	173,265
Number of indels	7,333	7,409	7,351
Insertion indels	2,961	2,992	3,164
Deletion indels	4,372	4,417	4,187
Large (> 10 bp) insertions	148	155	163
Large (> 10 bp) deletions	221	240	258
Number of hom ref loci	120,050	120,064	116,494
Number of hom var loci	220,731	221,029	224,711
Number of variant loci	220,731	221,029	224,711
Different		334,667	
Common		65,672

**Table 5:** Genes involved in lipid metabolisms with a moderate or high impact small variants common to the three

ID	Gene description	Function	VEP prediction
*Lipases & esterases*			
28726643	Secretory lipase	Enables triglyceride lipase activity	Start lost|Missense variants
28726875	Secretory lipase	Enables triglyceride lipase activity	Missense variants
28727692	Secretory lipase	Enables triglyceride lipase activity	Missense variants
28727697	Secretory lipase	Enables triglyceride lipase activity	Missense variants
28728326	Secretory lipase	Enables triglyceride lipase activity	Missense variants
28728420	Secretory lipase	Enables triglyceride lipase activity	Stop lost
28729307	Secretory lipase	Enables triglyceride lipase activity	Missense variants
28729308	Secretory lipase	Enables triglyceride lipase activity	Missense variants
28729753	Secretory lipase	Enables triglyceride lipase activity	Missense variants
28729754	Secretory lipase	Enables triglyceride lipase activity	Missense variants
28729086	Secretory lipase	Enables triglyceride lipase activity	Missense variant
28730532	Secretory lipase	Enables triglyceride lipase activity	Missense variants
28729104	GDSL family lipase	Hydrolase activity, acting on ester bonds	Missense variants
28729106	GDSL family lipase	Hydrolase activity, acting on ester bonds	Missense variants
28726738	Triglyceride lipase-cholesterol esterase	Lipid metabolic process	Missense variants
28726765	Phospholipase carboxylesterase family protein	Hydrolase activity	Missense variants
28726792	PLC-like phosphodiesterase	Phosphatidylinositol phospholipase C activity	Missense variants
28727315	PLC-like phosphodiesterase	Phosphoric diester hydrolase activity	Missense variants
28729016	PLC-like phosphodiesterase	Phosphoric diester hydrolase activity	Missense variants
28729037	Palmitoyl-protein thioesterase 1	Palmitoyl hydrolase activity and thioester hydrolase activity	Missense variants
28729456	Arylacetamide deacetylase	Hydrolase activity	Missense variants
28729458	Esterase lipase	Hydrolase activity	Missense variants
28726990	Acyl-thioesterase 2	Acyl-CoA hydrolase activity	Missense variants
*Sterol metabolism*			
28727161	*ERG5* (C-22 sterol desaturase)	Sterol metabolic process	Missense variants
28726452	*HMG1/2* (Hydroxymethylglutaryl-reductase)	Ergosterol biosynthetic process	Missense variants
28727558	*ERG13* (Hydroxymethylglutaryl-synthase)	Ergosterol biosynthetic process and farnesyl diphosphate biosynthetic process	Missense variant
28728578	*ERG24* (C-14 reductase)	Ergosterol biosynthetic process	Missense variants
28728633	Cytochrome-b5 reductase	Ergosterol biosynthetic process	Missense variant
28729490	*ERG2* (C-8 sterol isomerase)	Ergosterol biosynthetic process	Missense variant
28730546	*ERG9* (Farnesyl-diphosphate farnesyltransferase)	Ergosterol biosynthetic process and farnesyl diphosphate metabolic process	Missense variants
28729142	*ERG10* (Acetyl-CoA C-acetyltransferase)	Fatty acid beta-oxidation, sterol synthesis	Missense variant
28727196	Fatty acid hydroxylase	Ergosterol biosynthetic process	Missense variants
28729939	Hypothetical protein	Sterol binding	Missense variants
28730398	Isoprenoid biosynthesis-related protein	Farnesyl diphosphate biosynthetic process	7 Missense variants|1 Missense variant and splice region variant
*Phospholipid metabolism*			
28726906	Phospholipase C	Hydrolase activity, acting on ester bonds	Missense variants
28726907	Phospholipase C	Hydrolase activity, acting on ester bonds	Missense variants
28730308	Phospholipase C	Hydrolase activity, acting on ester bonds	Missense variants
28730591	Phospholipase C	Hydrolase activity, acting on ester bonds	Missense variants
28727150	SPO14-phospholipase D	Phosphatidylinositol binding, phospholipase D activity	Missense variants
28726795	Lysophospholipase PLB1	Phospholipase A2 activity	Missense variants
28728006	Lysophospholipase	Carboxylic ester hydrolase activity and palmitoyl-(protein) hydrolase activity	Missense variants
28728925	Cytosolic phospholipase	Glycerophospholipid catabolic process	Missense variants
28729637	Hypothetical protein	Sphingomyelin phosphodiesterase activity	Missense variants
28729683	Aminophospholipid transporting P-type ATPase	Phospholipid translocation	Missense variants
28730521	P-type ATPase (amino-phospholipid-translocase)	Phospholipid translocation	1 in-frame deletion | 7 Missense variants
28729006	Phospholipid-translocating ATPase	Phospholipid translocation	Missense variants
28728579	Hypothetical protein	Glycerophospholipid biosynthetic process	Missense variant & splice region variant | Missense variant
28728584	Phosphatidylserine decarboxylase PSD3	Phospholipid biosynthetic process	Missense variants
28726724	EPT1-sn-diacylglycerol ethanolamine cholinephosphotransferase	Phospholipid biosynthetic process	Missense variants
28727718	Phosphatidyl synthase	Cardiolipin biosynthetic process	Missense variants
28728940	Acyltransferase-domain-containing protein	Cardiolipin acyl-chain remodelling	Missense variants
28726864	Choline Kinase (*CKI1*)	Phosphatidylcholine biosynthetic process and phosphatidylethanolamine biosynthetic process	Missense variants
28727050	Patatin domain containing protein	Phosphatidylcholine metabolic process	Missense variants
28727422	Phosphatidylethanolamine n-methyltransferase (*CHO2*)	Phosphatidylcholine biosynthetic process	Missense variants
28727844	Myo-inositol monophosphatase	Inositol metabolic process	Missense variant
28729642	Phosphatidylinositol-4-kinase	Phosphatidylinositol phosphate biosynthetic process and phosphatidylinositol-mediated signalling	Missense variants
28728992	Vacuole-associated enzyme activator complex component	Phosphatidylinositol biosynthetic process	Missense variants
28728374	Hypothetical protein	Inositol phosphoceramide metabolic process	Missense variants
28728184	Nicotinate-nucleotide diphosphorylase	Phosphatidylinositol phosphate biosynthetic process	Missense variants
28727917	Atypical PIKK PI3K protein kinase	Phosphatidylinositol-3-phosphate biosynthetic process and phosphatidylinositol mediated signalling	Missense variants
28727417	Inositol hexakisphosphate kinase inositol pyrophosphate synthase	Inositol metabolic process and inositol phosphate biosynthetic process	Missense variant
28726664	Cdc4 and related F-box and WD40 protein	Phosphatidylinositol binding	Missense variants
28727015	Diadenosine hexaphosphate hydrolase	Diphosphoinositol polyphosphate metabolic process	Missense variants
28727024	1-phosphatidylinositol-3-phosphate 5-kinase	Phosphatidylinositol phosphate biosynthetic process	Missense variants
28727350	Saicar synthase-like protein	Phosphatidylinositol metabolic process	Missense variants
28726868	PX domain containing protein	Phosphatidylinositol binding	Missense variants
28726871	*AUR1* (Inositol phosphorylceramide synthase)	Inositol phosphoceramide synthase activity	Missense variant
*Fatty acid metabolism*			
28730094	17-beta-hydroxysteroid dehydrogenase	Fatty acid elongation	Missense variants
28730159	Hypothetical protein	Fatty acid biosynthetic process	Missense variants
28727736	Hypothetical protein	Fatty acid catabolic process and in triglyceride biosynthetic process	Missense variants
28728011	Peroxisomal acyl-CoA thioester hydrolase 1	Fatty acid catabolic process	Missense variants
28729494	Hypothetical protein	Fatty acid elongation, sphingolipid biosynthetic process and very long-chain fatty acid biosynthetic process	Missense variants
28728787	ATP-binding subfamily d member 2	Fatty acid beta-oxidation, long-chain fatty acid import into peroxisome and very long-chain fatty acid catabolic process	Missense variants
28726827	Acyl-CoA oxidase	Fatty acid beta-oxidation using acyl-CoA oxidase and lipid homeostasis	Missense variants
28727363	Acetyl-CoA synthetase-like protein	Long-chain fatty acid metabolic process	Missense variants
28726921	Acyl-CoA dehydrogenase	Fatty acid beta-oxidation, involved in medium-chain fatty acid catabolic process	Missense variants
28727005	Peroxisomal half ABC transporter	Fatty acid beta-oxidation, long-chain fatty acid import into peroxisome, and very long-chain fatty acid catabolic process	Missense variants
28729045	Acetyl-CoA acyltransferase 2	Fatty acid beta-oxidation	Missense variant
28728338	Acetyl carboxylase	Fatty acid biosynthetic process	Missense variants
28728375	Fatty acid elongase	Fatty acid elongation in monounsaturated, polyunsaturated and saturated fatty acid, in sphingolipid biosynthetic process, in very long-chain fatty acid biosynthetic process	Missense variant
28729115	ATP-citrate synthase	Fatty acid biosynthetic process	Missense variants
28729865	Polyketide synthase	Fatty acid biosynthetic process	Missense variants
28728046	Polyketide synthase	Fatty acid biosynthetic process	Missense variants
28729055	Δ12 fatty acid desaturase	Synthesis of polyunsaturated fatty acids	Missense variants
28729274	Δ9 desaturase	Synthesis of unsaturated fatty acids	Missense variant
28726876	Carnitine acyl carnitine carrier	Carnitine transmembrane transport	Missense variant
28728234	Acyltransferase CTAse COT CPT	Carnitine metabolic process	Missense variants
*Other genes related to lipid metabolism*			
28729152	Sphingosine hydroxylase	Lipid biosynthetic process	Missense variants
28727194	Protein of class 3 family	Lipid metabolic process	Missense variants
28726435	Acid sphingomyelinase	Hydrolase activity	Missense variants
28726734	ABC1 domain containing protein	Lipid homeostasis	Missense variants
28726824	Mitochondrial distribution and morphology protein	Lipid binding	Missense variants
28728172	Hypothetical protein	Lipid binding	Missense variant
28727246	Phosphatidate cytidylyltransferase	Phosphatidate cytidylyltransferase activity	Missense variants
28727719	Orm1 type endoplasmic reticulum protein	Ceramide metabolic process, intracellular sphingolipid homeostasis and negative regulation of ceramide biosynthetic process	Missense variants
28729010	Serine palmitoyltransferase	Ceramide biosynthetic process and sphingosine biosynthetic process	Missense variants
28728237	Glycerol-3-phosphate acyltransferase	Cellular lipid metabolic process	Missense variants
28729395	Glycosyltransferase family 57 protein	Oligosaccharide-lipid intermediate biosynthetic process	Missense variants
28727906	Glycosyltransferase family 4 protein	Oligosaccharide-lipid intermediate biosynthetic process	Missense variant

## Discussion

Due to the lack of the fatty acid synthase, *Malassezia* yeasts are unable to synthesize fatty acids de novo and rely on the environment as a main source. Thus, changes in the environment and its external fatty acid composition could represent a challenge for *Malassezia* yeasts [6, 8]. *Malassezia* species have a high number of enzymes related to the lipid metabolism to supply their lipid requirements. These enzymes may vary between species, conferring different metabolic versability to adapt to different environments [6, 8, 10, 29]. *Malassezia pachydermatis* is the only species within the genus able to grow on SGA without lipid supplementation, being a more versatile lipid-dependent yeast [8]. However, recently atypical *M. pachydermatis* strains that cannot grow on SGA have been observed [18–20]. To assess the basis of this atypical lipid dependency of some *M. pachydermatis* strains, the genome of three strains was sequenced and compared with the neotype strain. As the strains were unable to grown on SGA, they were cultured on SGA supplemented with Tween 20 and Tween 40. These lipid dependent *M. pachydermatis* strains have shown good growth on SGA with the addition of Tween 20 and Tween 40 [20] and they grew well in our study.

The analysis of the genome of these atypical *M. pachydermatis* strains revealed shared moderate to high impact SVs and small variants. These genetic variants were predicted to impact protein function, although the exact effects are not fully understood due to limited knowledge of the species' lipid metabolism. Although no SVs targeted lipid metabolism genes, a high-impact deletion was identified in the gene encoding the tripeptidyl peptidase enzyme. This enzyme may be involved in the degradation of peptides derived from peptone, a component of SGA known to contain fatty acids. The disruption of this enzyme could potentially alter the processing of peptone-derived substrates, thereby influencing fatty acid availability and metabolic responses in this medium [30].

Regarding small variants, we focused on those that affect lipid metabolism related genes. In our study, these atypical *M. pachydermatis* strains showed small variants in 23 lipase and esterase genes. The *M. pachydermatis* reference genome has 13 genes encoding secretory lipases, and in our strains, 12 out of these 13, showed moderate or high impact variants. Lipase genes are involved in the release of fatty acids from a variety of lipid compounds found in the environment, thus enabling lipid synthesis in *Malassezia* species [29, 31]. The two lipases showing high impact small variants were TGL3 and catalyze the hydrolysis of ester linkages of triglycerides [6]. The ability to uptake those fatty acids for their use in lipid synthesis is essential to sustain the growth of *Malassezia* [29].

Alterations were observed in genes encoding phospholipases, phosphodiesterases and other enzymes related to the phospholipid metabolism. Phospholipids are structural components of fungal cell membranes and play essential roles in their biology [7]. Moreover, phosphatidylcholine is an abundant glycerophospholipid found on eukaryotic membranes. In the presence of external choline, *M. pachydermatis* could synthetize phosphatidylcholine through both the Kennedy and the CDP-choline pathways [6, 7]. Within the genus *Malassezia, M. pachydermatis* is the only species able to synthesize phosphatidylcholine via the Kennedy pathway due to the presence of the choline kinase (*CKI1*) gene [6, 32]. However, our three atypical *M. pachydermatis* strains showed 5 moderated impact VCs in this gene, potentially altering phosphatidylcholine production through the Kennedy pathway. Nevertheless, another gene encoding a phosphatidylethanolamine n-methyltransferase (*CHO2* gene) related to the synthesis of phosphatidylcholine through the CDP-choline pathway showed ten moderate impact small variants. The depletion of phosphatidylcholine in yeasts has been linked to a shift in the fatty acid composition of the cells, leading to an increase of ratios of C16 over C18 and saturated over unsaturated acyl chains. This shifting relies on the fatty acid composition of the environment and the adaptation mechanisms of the yeasts [32]. These mechanisms of adaptation are available in yeasts with an intact fatty acid synthesis machinery as *Saccharomyces cerevisiae* [32], whereas our atypical *M. pachydermatis* strains may face some difficulties adapting to the growth in a media without lipid supplementation.

Additional small variants were found in genes essential for fatty acid metabolism in our atypical *M. pachydermatis* strains. Twenty altered genes affected fatty acid synthesis, elongation, degradation, and transport, suggesting reduced ability to utilize environmental fatty acids. Within this group of genes, the gene encoding for the Δ9-desaturase responsible for the synthesis of unsaturated fatty acids showed moderate impact small variants in the three atypical strains. This enzyme converts palmitic and stearic acids into palmitoleic and oleic acids, which are essential for membrane fluidity and growth. *Malassezia globosa* and *Malassezia restricta* lack this enzyme and rely entirely on external unsaturated fatty acids, so its alteration in atypical strains may reduce metabolic versatility [9, 11, 31]. Similarly, the Δ12-desaturase gene, involved in polyunsaturated fatty acid synthesis, showed two moderate impact variants [33]. It has been demonstrated that in *Malassezia*, growth is best stimulated by unsaturated fatty acids [10], so these changes may reduce metabolic versatility and could explain the need for additional lipid supplementation compared to the neotype strain. Furthermore, the polyketide synthase gene, which is structurally similar to fatty acid synthase and may contribute to lipid assimilation versatility, also showed moderate alterations [8, 29]. This could indicate a reduced ability to utilize certain environmental fatty acids and adapt to changing conditions.

In conclusion, sequencing the genome of three atypical lipid-dependent *M. pachydermatis* strains allowed a better understanding of this species and the growth differences observed between strains. The analysis of the small variations observed in genes related to lipid metabolism such as lipases, genes related to phospholipid metabolism and fatty acid metabolism suggested a variation in their ability to adapt to environmental changes and their requirements to grow in different culture media. We emphasize the small variations identified in 12 of the 13 genes encoding secretory lipases, as well as in the *CKI1* gene, which is unique to *M. pachydermatis* within *Malassezia* genus. Thus, possibly explaining the lack of growth observed in SGA as they might not be able to utilize the lipid fractions within its peptone component and require additional lipid supplementation. Nevertheless, more studies would be necessary to further understand the lipid versatility of *M. pachydermatis* at the transcriptional level.

Finally, establishing genotype-phenotype-pathogenicity correlations and assessing host interaction and disease potential are critical next steps to take in future studies. However, it is important to note that the isolation of these atypical strains is extremely rare [20]. In our collection of more than 1,500 *M. pachydermatis* isolates, only three have shown this atypical lipid-dependent phenotype, and all were recovered from clinically healthy animals. This rarity suggests that these strains may represent uncommon auxotrophic mutants rather than a widespread pathogenic form, but further research is needed to clarify their biological significance.

## Data Availability

The genome resequencing information of M. pachydermatis MA366, MA374, and MA380 have been deposited in the NCBI Sequence Read Archive (SRA) database under accession PRJNA1293882.
